# Methotrexate Restores CD73 Expression on Th1.17 in Rheumatoid Arthritis and Psoriatic Arthritis Patients and May Contribute to Its Anti-Inflammatory Effect through Ado Production

**DOI:** 10.3390/jcm8111859

**Published:** 2019-11-03

**Authors:** Marion Bossennec, Céline Rodriguez, Margaux Hubert, Anthony Di-Roio, Christelle Machon, Jérôme Guitton, Priscilla Battiston-Montagne, Mathilde Couturier, Hubert Marotte, Christophe Caux, Fabienne Coury, Christine Ménétrier-Caux

**Affiliations:** 1 Immunology Department, University of Lyon, Claude Bernard Lyon 1 University, INSERM 1052, CNRS 5286, Cancer Research Center of Lyon (CRCL), Centre Léon Bérard, 69008 Lyon, Francechristophe.caux@lyon.unicancer.fr (C.C.); 2 Team “Therapeutic Targeting of the Tumor Cells and Their Immune Stroma”, INSERM U1052, CRCL, 69008 Lyon, France; 3 Analytical Chemistry Department, ISPB Faculty of Pharmacy, University of Lyon, Claude Bernard Lyon 1 University, 69008 Lyon, France; 4 Biochemistry and Toxicology Laboratory, Lyon Sud Hospital, 69310 Pierre-Bénite, France; 5 Toxicology Department, ISPB Faculty of Pharmacy, University of Lyon, Claude Bernard Lyon 1 University, 69008 Lyon, France; 6 Cytometry Platform, CRCL, 69008 Lyon, France; 7 Rheumatology Department, Lyon Sud Hospital, 69310 Pierre-Bénite, France; docteurcouturierrhumato@gmail.com (M.C.);; 8 Rheumatology Department, CHU Saint-Etienne, 42100 Saint-Etienne, France; hubert.marotte@chu-st-etienne.fr; 9 INSERM U1059, Jean Monnet University, 42100 Saint-Etienne, France; 10 INSERM, UMR 1033, Claude Bernard Lyon 1 University, 69008 Lyon, France

**Keywords:** Th1.17, IL-17A, IFN-γ, CD73, adenosine, rheumatoid arthritis, psoriatic arthritis, methotrexate, regulation

## Abstract

Objectives: Th1.17 are highly polyfunctional, potentially harmful CD4^+^ effector T cells (Teff) through IFN-γ and IL-17A coproduction. Th1.17 take part in the pathophysiology of rheumatoid arthritis (RA) and psoriatic arthritis (PsA), in which their hyper activation results in part from defects in negative regulation mechanisms. We recently demonstrated that the ecto-nucleotidase CD73 delineates a Th1.17-enriched Teff population and acts as an endogenous regulatory mechanism. Because Methotrexate (MTX), used as first line treatment of RA and PsA, increases extracellular concentrations of AMP and immunosuppressive adenosine, we investigated the modulation of CD73 by MTX treatment on Teff in RA/PsA patients. Methods: In a prospective cohort of 26 RA and 15 PsA patients before or under MTX treatment, we evaluated CD73 expression on blood Teff subsets, their cytokine production and AMPase functions. Results: We showed a decreased CD73 expression on Th1.17 and Th1 in untreated patients compared to healthy donors that was partly restored under MTX. This decrease in untreated patients leads to a halved Ado production by Th1.17 cells. CD73^+^ Teff remained functional under MTX treatment, but their CD73 re-expression may contribute to control their activation. Conclusion: Our study unveils uncovered mode of action of MTX on Teff subsets modulation and in the adenosine-dependent termination of inflammation in RA and PsA.

## 1. Introduction

Rheumatoid Arthritis (RA) and Psoriatic Arthritis (PsA) are chronic inflammatory disorders characterized by tenderness and swelling of the joints. If not treated, both can lead to bone erosion, resulting in joint destruction due to osteoclasts activation. RA is an autoimmune systemic disease characterized by the presence of anti-citrullinated protein antibodies (ACPA) and rheumatoid factor (RF) [1]. PsA is usually associated with the skin condition psoriasis (Pso) [2]. In both cases, study of the immune infiltrate of inflamed joints has shed light on particularly deleterious memory CD4^+^ T helper lymphocytes (Th): Th17 and Th1.17 [3,4,5]. These cells, found enriched at the inflamed site, secrete high amounts of the pro-inflammatory cytokine IL-17A, responsible for many features of both RA and PsA. IL-17A indeed contributes to the recruitment of pro-inflammatory monocytes and neutrophils at the site of inflammation, and promotes the transformation of the fibroblast-like synoviocytes present in the synovial lining toward a pro-inflammatory phenotype [6,7,8]. In the presence of IL-17A, these cells secrete IL-6 and TNF [9] and high levels of RANKL, which in turn stimulate osteoclast differentiation [10]. Therefore, IL-17A is implicated in the positive feedback loop at the roots of the chronic inflammation and bone destruction observed in joints. Moreover, in RA and PsA, T cells display reduced sensitivity to the diverse immune suppressive functions exerted by regulatory T cells (Treg) [11]. Treg are involved in the modulation of extracellular purine derivatives levels, through their membrane expression of the ecto-enzyme CD39, which degrades pro-inflammatory extracellular ATP into AMP. AMP can, in turn, be degraded into immunosuppressive adenosine (Ado) by the ecto-nucleotidase CD73. We recently demonstrated that in human, Treg express CD39, but no CD73; therefore, third-party CD73 expressing cells are required for the degradation of AMP into Ado [12]. CD73 is in particular expressed by a fraction of non-regulatory CD4^+^ memory T cells (Teff) strongly enriched in Th1.17 and Th17 [12]. We showed that CD73 expression by Teff renders them selectively sensitive to Ado through their cooperation with CD39^+^ Treg for extracellular ATP degradation. In the context of chronic inflammation, modulation of expression of these ecto-enzymes on Teff, and thus of Ado production, may regulate T cell activation. 

RA and PsA treatment options are mainly composed of an arsenal of disease modifying anti rheumatic drugs (DMARDs), among which Methotrexate (MTX) is still largely used due to its efficiency, low toxicity and cost effectiveness [13,14]. Its mode of action at low doses (5–20 mg/week) for RA and PsA treatment remains incompletely understood. Apart from its role as anti-folate agent, other mechanisms have been proposed to explain clinical improvement upon MTX treatment (reviewed in [13]). Robust evidence suggests that MTX acts through the potentiation of Ado signaling. Indeed, MTX inhibits the 5-Aminoimidazole-4-carboxamide ribonucleotide (AICAR) transformylase enzyme (ATIC) [13,14,15] resulting in increase in both intracellular AICAR levels and extracellular AMP and Ado release. However, the majority of extracellular Ado is in fact generated from extracellular transport of ATP and AMP degraded by CD39/CD73 that are required for MTX-induced immune suppression in mouse models [16]. Extracellular Ado inhibits T cell activation and proliferation in a paracrine manner, through the engagement of its A2A and A2B receptors which expression is upregulated on activated Teff [12,17,18], and through the amplification of Treg expressing A2AR [19].

In this study, we evaluated the presence and distribution of CD73^+^ Teff among Th populations in the blood of untreated RA and PsA patients. We also assessed the functionality of these cells in patients in comparison to healthy donors’ blood. We demonstrated that loss of CD73 membrane expression in untreated patients resulted in a lowered Ado production, potentially contributing to impaired control of the inflammation. Under MTX treatment, CD73 was partially recovered on Teff, which was associated with a restored capacity to degrade AMP into Ado. 

Therefore, CD73 expression level on Teff may represent a therapeutic target worth considering in the treatment of RA by restoring and stabilizing it through an immunosuppressive feedback loop enabling Ado production.

## 2. Experimental Section

### 2.1. Patients

Patients aged ≥ 18 years, naive of biologics, with RA fulfilling the American College of Rheumatology and European League Against Rheumatism 2009 criteria [20] or with PsA fulfilling the Classification of PsA (CASPAR) criteria [21] were enrolled in the LADORIC study (NCT03953378) after written informed consent, in accordance with the Declaration of Helsinki. The approval of the ethics committee was not required in accordance with our institution’s policy. Clinical and biological information was collected prospectively (Table 1). 

Severity of the disease was assessed using the Disease Activity Score in 28 joints (DAS28-CRP) [22] for RA and CRP for PsA at baseline visit and 3 or 6 months after MTX treatment onset. DAS28-CRP ≥ 3.2 in untreated RA patients and CRP ≥ 6 mg/mL in untreated PsA patients were set as thresholds for active disease. Samples of venous blood and, when available, synovial fluid (SF) were collected for each patient before the onset of MTX (untreated patients) or during the course of MTX treatment (MTX-treated patients). Blood samples from age- and sex-matched anonymous healthy donors (HD) were obtained from the Etablissement Français du Sang.

### 2.2. Peripheral Blood Mononuclear Cells (PBMC) and Synovial Fluid Mononuclear Cells (SFMC) Isolation 

Blood samples were centrifuged on a Ficoll (Eurobio, Les Ullis, France) density gradient to purify PBMC. SF samples were diluted in HBSS (Life Technologies, Cailloux sur Fontaines, France) containing 10 mM EDTA (Sigma, Saint Quentin-Fallavier, France), and processed as blood samples to isolate SFMC.

### 2.3. Flow Cytometry Analyses

Multi-parametric Flow Cytometry (FC) staining were performed on PBMC or SFMC from RA and PsA patients using different panels described in Table S1 (Panels A and B) to assess CD73 expression on total memory T cells and within Th subsets. When specified, proliferation was assessed using an anti-human anti-Ki67 antibody (Ki-67, Biolegend, Saint-Cyr-l’Ecole, CA, France). For FoxP3 intracellular staining to characterize Treg, cells were treated using the FoxP3 Fixation and Permeabilization kit (Life Technologies), according to manufacturer instructions. Samples were analyzed on a LSR-Fortessa (BD Biosciences, Pont de Claix, France) with conserved settings throughout the entire study. Data were analyzed using FlowJo Software (Tree Star v10.4, Franklin Lakes, NJ, USA). 

### 2.4. Analysis of Cytokines Production Capacity after Reactivation

PBMC were activated with PMA and Ionomycin (Sigma-Aldrich) as previously described [12] and intracellular cytokines (IL-17A, TNF-α, IFN-γ, IL-22) produced by CD73^+^ and CD73^neg^ Teff were analyzed using the specific panel described in Table S1 (Panel C). Stainings were analyzed on a LSR-Fortessa and Teff polyfunctionality was evaluated using the Boolean method (FlowJo software) and then processed using Pestle and SPICE v5.3 softwares, National Institute of Allergy and Infectious Diseases, Bethesda, MD, USA.

### 2.5. CD73^+^ Teff Sorting and In Vitro Activation to Asses CD73 Dynamic Expression

Memory CD4^+^ T cells were purified from HD PBMCs using MagniSortTM Human CD4^+^ Memory T Cell Enrichment Kit (LifeTechnologies). CD73^+^CD4^+^ Teff (CD4^+^CD45RA^neg^CD127^+^CD25^neg^CD39^neg^ CD73^+^) and CD73^neg^CD4^+^ Teff (CD4^+^CD45RA^neg^CD127^+^CD25^neg^ CD39^neg^CD73^neg^) were sorted from purified memory CD4^+^ T cells by multi-parametric FC (FACSAria III, BD Biosciences) using antibodies against CD25 (2A3, BD-Biosciences), CD45RA (2H4LDH11LDB9, Beckman-Coulter, Brea, CA, USA), as well as CD127 (eBioRDR5), CD39 (eBioA1) and CD73 (AD2) (all from e-Bioscience), alongside a viability marker (DAPI). 

Sorted populations were stained with CellTrace Violet (CTV) (20 μM, LifeTechnologies) proliferation markers before incubation with Expand beads (LifeTechnologies) (ratio 1:4) in 96-round-bottomed-well plates in 200 μL of complete RPMI medium for 4 days at 37 °C under 5% CO_2_.

After 4 days’ proliferation, part of the cells was stained for viability and CD73 expression with anti-human anti-CD73 and cells were fixed with 2% Formaldehyde solution (Sigma) and stored at 4 °C. The rest of the cells were washed and removed from Expand beads using a magnet. They were then incubated for a 2-day period without TCR signal in 200 μL of complete RPMI medium containing IL-2 (50 UI, Chiron). Cells were then stained for viability and CD73 expression as well. All time points were analyzed concomitantly by FC for viability, proliferation and CD73 expression (LSR Fortessa, BD Biosciences). 

### 2.6. Th1.17, Th1 and Th17 Sorting for In Vitro AMP Degradation Assay

Memory CD4^+^ Teffs were purified using MojoSort Human CD4^+^ Memory T Cell Isolation Kit (Biolegend). Th1, Th17 and Th1.17 cells were sorted from purified memory CD4^+^ T cells by multi-parametric FC (FACSAria III, BD Biosciences) based on chemokine receptors expression [23] (Figure S1) using the combination of antibodies described in Table S1 (Panel D). 

The capacity of 5 × 10^4^ cells of each Th subpopulation of HD, RA untreated or MTX-treated patients to degrade AMP was analyzed after 2 h incubation at 37 °C under 5% CO_2_ with labeled AMP (AMP_13C,15N_, 37.5 µM, Sigma-Aldrich) in 200 μL of serum-free RPMI medium supplemented with antibiotics and L-glutamine (Life Technologies). As a control, cells were pre-incubated with a CD73 chemical inhibitor (APCP, 50 μM, Sigma) for 30 min before adding AMP_13C,15N_ to assess CD73-specific degradation. 

### 2.7. Nucleotides and Nucleosides Quantification by Liquid Chromatography Coupled with Tandem Mass Spectrometry (LC-MS/MS)

Cell supernatants were harvested, boiled at 65 °C for 5 s and frozen at −20 °C. AMP_13C,15N_ and Ado_13C,15N_ were quantified in 50 μL of supernatant, after solid phase extraction, using a LC-MS/MS method as described [12,24]. Concentrations of nucleotides in the supernatants were calculated using calibration curves of the corresponding labeled nucleotides (AMP_15N_ and Ado_13C_). AMP_13C_ and Ado_15N_ were used as internal standards. We also verified that APCP did not interfere with AMP and Ado quantification.

### 2.8. Statistical Analysis

Data formatting and statistical tests were performed using Prism software (Graphpad Inc., San Diego, CA, USA). Kruskal-Wallis test, Mann-Whitney test and ANOVA2 were used when comparing unpaired data according to the parameters considered. Wilcoxon test and ANOVA2 with paired values were performed to analyze patients’ data follow up. We evaluated correlation between biological parameters and activity of the disease (DAS28-CRP) using Spearman correlation tests.

## 3. Results

### 3.1. Activated Th1.17 from Peripheral Blood of Untreated RA and PsA Patients Express Low Levels of CD73

**Figure 1:**
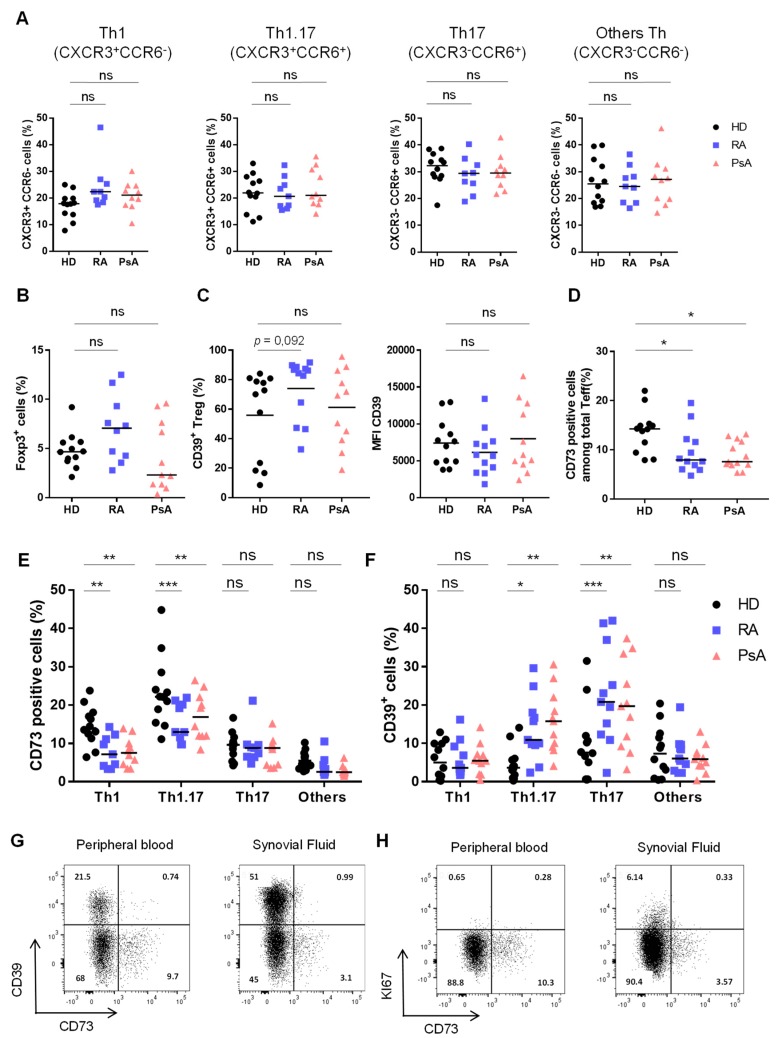
Unchanged Th populations but impaired CD73 expression on Th1 and Th1.17 from RA and PsA patients. (**A**). Th subsets frequencies based on phenotypic analysis (CXCR3/CCR6 staining) among memory CD4^+^ T cells. (**B**). Frequencies of FoxP3^+^ Treg among memory CD4^+^ T cells. (**C**). Frequencies (left) and MFI (right) of CD39^+^ Treg among memory CD4^+^ T cells. (**D**). CD73 expression on total Teff. (**E**). CD73 expression by Th subsets. (**F**). CD39 expression by Th subsets. (from A to F: analyses performed on peripheral blood). (**G**,**H**). Flow cytometry plots on total Teff in peripheral blood and SF of an untreated RA patient showing CD73/CD39 (**G**) and CD73/Ki67 (**H**) staining. (**A**–**D**): Kruskal-Wallis test, (**E**,**F**): ANOVA-2. * *p* < 0.05, ** *p* < 0.001, *** *p* < 0.0001.

Total memory CD4^+^ and CD8^+^ T cells frequencies were not modified in peripheral blood of untreated RA and PsA patients compared to HD (Figure S2A), neither were frequencies of Th subpopulations based on their CCR6 and CXCR3 expression [23] (Figure 1A). Treg frequency was unaltered in untreated RA and PsA patients’ blood (Figure 1B). However, in RA patients, Treg displayed a higher frequency of CD39 expression compared to HD and PsA patients, although the mean fluorescence intensity (MFI) of CD39 was not different (Figure 1C). Interestingly, we found a significantly lower CD73 expression on total Teff population from RA and PsA patients compared to HD (Figure 1D). Since we established that, in HD, CD73 is enriched in Th1.17 and Th17 phenotype [12], we stratified our analysis on Th subpopulations based on CXCR3 and CCR6 expression that allows to distinguish Th subsets with different cytokine pattern (Figure S1). CD73 expression was significantly decreased on Th1.17 (by 1.5 and 1.4 fold on RA and PsA patients respectively) and Th1 (by 1.9 fold on both RA and PsA patients) (Figure 1E).

Using CD39 [25,26], we demonstrated a significantly increased activation of blood-associated Th1.17 and Th17 in RA and PsA patients compared to HD ones (Figure 1F). In parallel, we observed that in vitro TCR stimulation was sufficient to decrease CD73 expression in sorted CD73^+^ Teff from HD (Figure S2B). Taken together, these results indicate that loss of CD73 expression on Th1.17 associated to high CD39 expression in untreated RA and PsA patients’ blood could reflect the activated state of this population. The access to paired samples of PBMC and SFMC for RA and PsA patients before treatment enabled us to show that the decrease of CD73 expression and CD39 up-regulation on total Teff was even more dramatic at the site of inflammation both in RA (Figure 1G) and in PsA (Figure S2C). In addition, proliferating cells (identified as Ki67^+^ cells) were included within CD39^+^CD73^neg^ Teff in RA (Figure 1H and Figure S2E) and PsA (Figure S2D,F) SF, further highlighting the absence of CD73 expression by proliferating Teff.

In untreated RA patients, percentage of CD39^+^ Treg positively correlated (*r* = 0.68, *p* = 0.03) with the severity of the disease evaluated through DAS28-CRP score. In contrast, no correlation was noticed for CD39 percentage on Th1, Th1.17 and Th17 subsets (not shown). No correlation with the severity of the disease was noticed for global CD73 expression on Teff nor global Th1, Th1.17 and Th17 proportions in blood. Moreover, the proportion of CD73 expressed by Th1 and Th1.17 populations did not correlate with disease severity despite they were strongly reduced in untreated RA patients compared to HD. In contrast expression of CD73 on Th17 and IL-17A production by CD73^+^ Teff tend to inversely correlate with disease severity (*r* = −0.65, *p* = 0.06 and *r* = −0.63, *p* = 0.07, respectively) (Figure S3). For PsA untreated patients no correlation was observed probably because either the heterogeneity of the disease or too small cohort size to achieve robust correlations.

### 3.2. Untreated RA and PsA Patients’ Blood Teff are Polyfunctional but Express Lower Levels of CD73 among IFN-γ/IL-17A Expressing Cells

**Figure 2:**
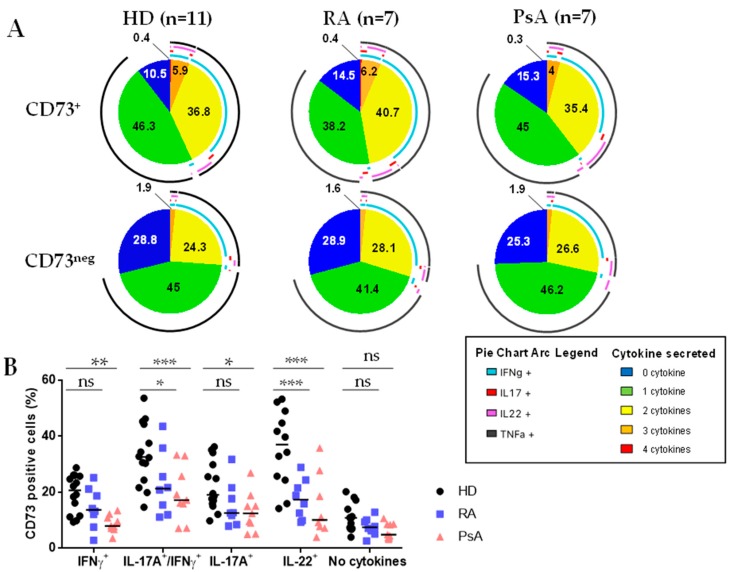

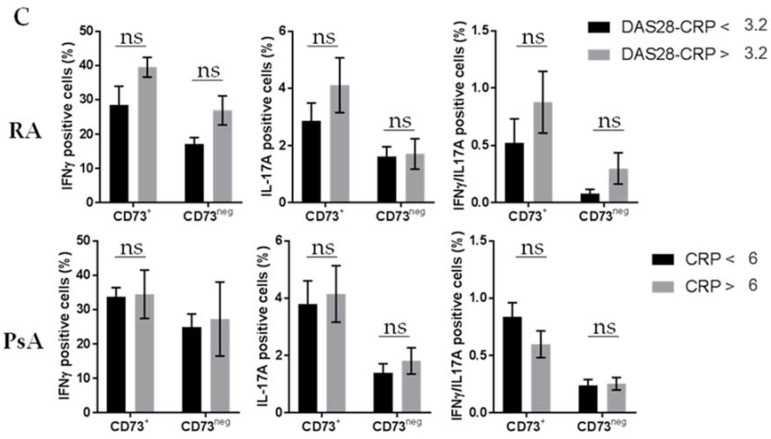
Polyfunctionality of Teff is not altered in RA and PsA patients but there are less CD73^+^ cells identified in IL-17A/IFN-γ secreting Teff. (**A**): SPICE^®^ representation of the cytokines secreted by either CD73^+^ or CD73^neg^ Teff from peripheral blood of HD, or untreated RA and PsA patients. (**B**): CD73 expression on Teff according to their secretion of IFN-γ, IL-17A and IL-22 in peripheral blood of HD or untreated RA and PsA patients. (**C**): IFN-γ and IL-17A mono- or co-production by Teff according to their CD73 expression in untreated RA and PsA patients stratified on DAS28-CRP score and CRP seric level respectively. (**B**,**C**): ANOVA-2. * *p* < 0.05, ** *p* < 0.001, *** *p* < 0.0001.

We previously showed that CD73 marks polyfunctional Teff in blood but also in healthy (tonsil, colon) and tumor (breast and ovarian) tissues [12]. In our setting, no striking modifications of the polyfunctionality (IFN-γ, IL-17A, and TNF-α) of CD73^+^ and CD73^neg^ Teff was noticed in untreated RA and PsA patients compared to HD (Figure 2A) and global levels of each cytokine were unchanged (Figure S4A). Of note, CD73^+^ Teff, which were higher single IL-22 producers in HD [12] were an even more important source of IL-22 in PsA (9.1 ± 2.6% versus 4.2 ± 1.3% of CD73^+^ Teff) (Figure S4B). In line with phenotypic analyses (Figure 1E), the IFN-γ/IL-17A coproducing cells, corresponding to Th1.17 (Figure S1), expressed significantly less CD73 (Figure 2B). Finally, Teff from untreated RA and PsA patients according to their DAS28-CRP or CRP level, respectively, showed enhanced IFN-γ and IL-17A production by CD73^+^ Teff in patients with active RA (DAS28-CRP ≥ 3.2), although not reaching statistical significance (Figure 2C).

### 3.3. Treatment of Patients with MTX Partially Restores CD73 Expression on Teff Resulting in an Enhanced Ado Production

**Figure 3:**
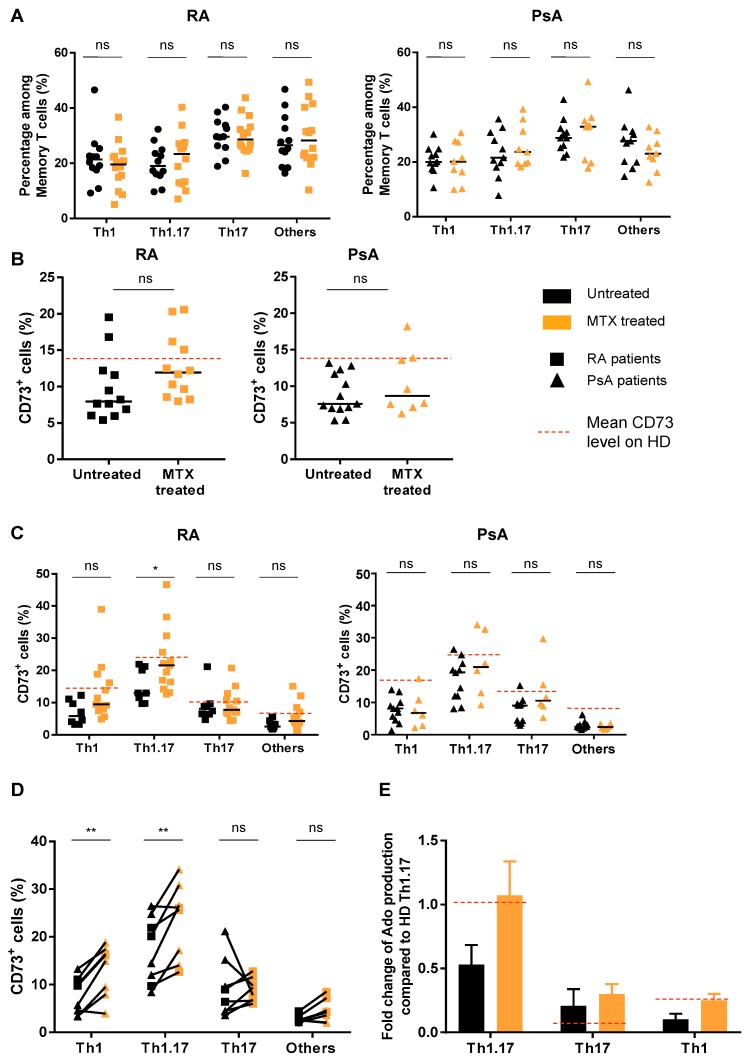
MTX treatment restores CD73 levels on Teff populations in RA patients but not in PsA patients. (**A**) Frequencies of Th subsets in peripheral blood of untreated versus MTX-treated RA (left) and PsA (right) patients. (**B**) CD73 percentages on total Teff in peripheral blood of untreated versus MTX treated RA (left) and PsA (right) patients. (**C**) CD73 percentages on Th subsets of untreated versus MTX-treated RA (up) and PsA (down) patients. (**D**) Paired samples showing CD73 percentages modulation in Th subsets upon MTX treatment in RA (*n* = 4) and PsA (*n* = 7) patients. (**E**) Fold change of Ado produced by sorted Th1.17, Th17 and Th1 subsets from untreated (*n* = 3) and MTX-treated RA (*n* = 3) patients. Cells were incubated for 2 h with AMP_13C15N_ isotope (37.5 µM) +/− APCP (50 µM) before Ado quantification in supernatants by LC-MS/MS. Ado production was normalized to the production by Th1.17 from HD’s blood used as reference. **A** and **C**: ANOVA-2, **B**: Mann-Whitney test, **D**: ANOVA-2 with paired values. * *p* < 0.05, ** *p* < 0.001.

Due to the described MTX involvement in the regulation of purine metabolism [13,26], we also analyzed the impact of MTX treatment on CD73 expression by Th subpopulations. We recently demonstrated that exogenously added Ado on CD73^+^ Teff blocked their proliferation and cytokine secretion [12]. Here, we evidenced that CD73^+^ Teff re-acquired CD73 expression after two days in resting condition (no TCR signaling), demonstrating dynamic CD73 protein expression at the surface of Teff (Figure S2B). In this context, we analyzed CD73 expression on Th subsets in MTX-treated RA and PsA patients, considering that a gain of CD73 expression could reflect drug efficiency on these populations and could contribute to its anti-inflammatory effects. MTX did not impact Th subsets frequencies compared to untreated RA and PsA patients (Figure 3A). However, CD73 expression on Teff was increased in MTX-treated RA patients compared to untreated ones while no significant variation was observed in PsA patients (Figure 3B). Interestingly, in RA patients, CD73 expression was especially increased within Th1.17 (Figure 3C) and overall CD73 expression level tended towards those observed in HD. Finally, the analysis of paired patients’ blood samples (for, respectively, four and seven patients for RA and PsA) before and under MTX treatment showed significantly increased CD73 levels on Th1 and Th1.17 subsets (Figure 3D), the two populations with significantly altered CD73 expression in untreated patients compared to HD (Figure 1E). This impact of MTX was restricted to CD4^+^CD73^+^ Teff, as we did not notice striking variations in memory CD8^+^ T cell frequencies (Figure S5A) or in Treg or CD39^+^ Treg frequencies (Figure S5B,C) even in paired samples (Figure S5D–F). Of note, CD39 levels did not seem to be modulated by MTX treatment on Teff either (Figure S5G,H). 

Among HD donors’ Th subsets, Th1.17 was the main subset able to degrade AMP into Ado (Figure S6), in line with its strongest expression of CD73 (Figure 1E). This generation of Ado was dependent on CD73, as demonstrated by the loss of Ado in the presence of APCP (Figure S6). In comparison the quantities of Ado generated was four-fold lower for the Th1 subset. Interestingly, we evidenced that reduced CD73 expression on Th1.17 and Th1 of untreated RA patients was associated with 50% decrease in Ado production (Figure 3E). Strikingly, upon MTX treatment, the level of Ado production was restored to HD donor levels (Figure 3E). 

### 3.4. MTX Treatment Impacts Teff Polyfunctionality

**Figure 4:**
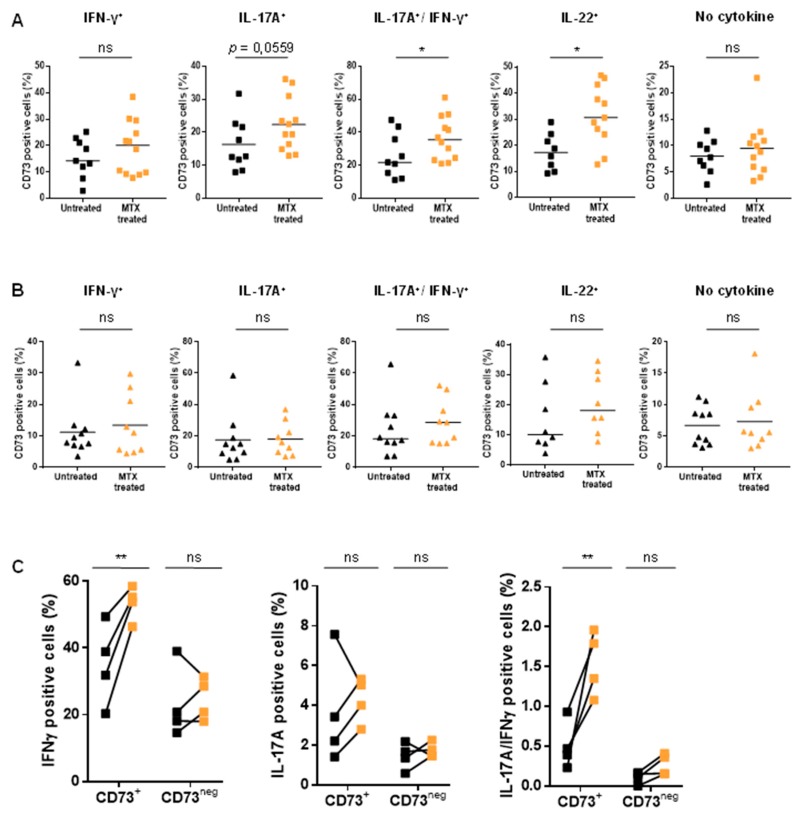

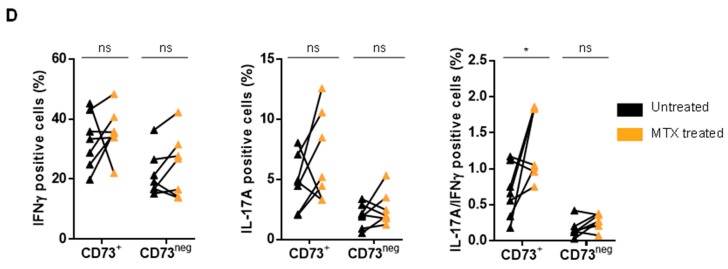
Impact of MTX treatment on Teff polyfunctionality. (**A**,**B**). Analysis of CD73 expression on cytokine secreting Teff mirrors phenotypic analysis in RA (**A**) and PsA (**B**) patients. (**C**,**D**). IFN-γ, IL-17A producing and IFN-γ/IL-17A co-producers in RA (*n* = 4) (**C**) and PsA (*n* = 7) (**D**) patients before initiation of MTX treatment and under MTX treatment. **A**,**B**: Mann-Whitney tests, **C**,**D**: ANOVA-2 paired samples * *p* < 0.05, ** *p* < 0.001.

Interestingly, we showed that CD73 expression was increased on cells coproducing IFN-γ and IL-17A, corresponding to Th1.17, from MTX-treated RA patients (Figure 4A,B). These results mirror the phenotypic analysis obtained for the Th1.17 subset (Figure 3C). In addition, we evidenced that MTX treatment significantly increased CD73 frequency among IL-22 producing cells in RA patients (Figure 4A). Of note, no major variation on global pattern of cytokines production was detected in RA patients when comparing untreated and MTX-treated samples (Figure S7A) and a rather modest increase of IL-17A^+^ and IFN-γ^+^/IL-17A^+^ coproducing cells was observed in MTX-treated PsA patients compared to untreated ones (Figure S7B). CD73^neg^ Teff functionality was not altered by MTX neither in RA nor in PsA patients (Figure 4C,D). Surprisingly, CD73^+^ Teff displayed increased capacity to produce IFN-γ in RA patients (Figure 4C) and to co-produce IFN-γ and IL-17A after MTX treatment in both RA (Figure 4C) and PsA patients (Figure 4D).

## 4. Discussion

In this study, we demonstrated a dynamic expression of CD73 on Th populations in peripheral blood of RA and PsA patients modulated by MTX treatment. Decreased CD73 levels were detected on Th1, Th1.17, Th22 and to a lesser extent on Th17 cells in untreated patients compared to HD. The partial restoration of CD73 levels on these Th effectors upon MTX treatment reveals a novel regulatory action of this immunosuppressive drug. 

The development of new therapeutic approaches for the treatment of RA and PsA is an ongoing challenge that requires to decipher the pathologic modifications of immune cells at the roots of the establishment of the chronic inflammatory state that characterizes these pathologies. 

The diverse pro-inflammatory Th populations appear as good targets to breakdown the chronic inflammation [27]. In the context of systemic inflammation, the monitoring of CD39/CD73 expression levels in the peripheral blood of patients may therefore be a marker of Teff inflammatory potential, an important parameter in the evaluation of disease activity. We focused our work on the polyfunctional CD73^+^ Teff population that we recently described in human as enriched in Th1.17 and Th17 [12]. Since we previously showed that CD73 expression renders them selectively sensitive to the inhibition by CD39^+^ Treg through autocrine Ado activity, we aimed at better understanding the dynamic of CD73 expression on Th1.17 and Th17 that play a central role in RA and PsA [3,4,28]. FC analyses on PBMC from untreated RA and PsA patients did not highlight any modification in Treg frequencies, consistent with previous data [29]. However, we noticed up-regulated expression of CD39 on these Treg in untreated RA, which was correlated to disease activity at odds to other studies focusing on RA [3,28,30]. CD73 expression was significantly diminished on blood Teff in untreated RA and PsA patients compared to HD. This decrease was even stronger in SFMC, indicating that blood CD73 levels might mirror the extent of the inflammation in the affected joints as previously suggested in juvenile idiopathic arthritis [31]. Dampened or inefficient Treg suppressive functions described by others in RA and PsA patients [32] could partly result from this decreased expression of CD73 on activated Teff, as it may impair CD39^+^ Treg and CD73^+^ Teff cooperation for self-inhibition through autocrine Ado production that will act only in a nearby environment due its very short half-life. In line with this, CD73 expression level on synovial lymphocytes was proposed as a marker of disease activity in idiopathic juvenile arthritis [31] and could also be monitored in adults. 

This decreased CD73 expression was particularly pronounced on Th1 and Th1.17 subsets compared to HD. Considering the strong plasticity of Th differentiation and recent data showing that Th1.17 can shift to unconventional Th1 cells when exposed to inflammatory cytokines [3,33], we suggest this decreased CD73 expression observed on Th1 might comprise activated non-classical Th1 cells phenotypically characterized by CXCR3 expression. Interestingly, this loss of CD73 expression was associated with a decreased production of Ado by Th1.17 and Th1 cells from untreated patients. Therefore, we propose that loss of CD73 expression on Teff might be a mechanism of escape to the suppression exerted by Treg, thereby enabling them with uncontrolled proliferation and secretion of pro-inflammatory cytokines. Although not reaching statistical significance, CD73 downregulation was also observed on Th17 cells that highly express CD39, suggesting an activated state. In addition, the only parameter that negatively correlated to disease activity in RA was CD73 expression by Th17 cells. This suggests that CD73 expression on Th17 cells could contribute in RA to limit the aggressiveness of the disease through the generation of Ado that could impair their own expansion but not IL-17A production as we previously demonstrated the inability of Ado to alter IL-17A secretion in contrast to other cytokines (IFN-γ, GM-CSF, IL-22, IL-10, IL-13) [12,30]. 

Considering the polyfunctionality of Teff in untreated patients, we did not notice significant variations compared to HD in contrast to other studies reporting higher frequency of IL-17A producing cells in peripheral blood of PsA patients [34,35]. These discrepancies may rely on the methodology used to investigate IL-17A production, and also from patients’ medical history and sampling criteria. These results remain however to be confirmed in a bigger cohort of untreated RA and PsA patients. It would also be interesting to confirm that Teff from untreated patients display low or no alteration of their cytokine production in presence of exogenous AMP because of their low expression of CD73 contrary to HD [12]. In addition, we evidenced that the frequency of single IL-22 producing cells is higher in untreated PsA patients compared to HD and to untreated RA patients (Figure S4B). This is in line with the high IL-22 production by Teff previously reported in blood of PsA patients [36]. CD73 expression could not precisely investigated on Th22 cells using as specific phenotypic markers are lacking so far, but we demonstrated that single IL-22-producing cells were enriched among CD73^+^ Teff compared to CD73^neg^ counterparts, although this population was decreased in untreated patients. While IL-22 has been reported to contribute to Pso pathogenesis [37], there is, however, no clear consensus as to whether this cytokine is deleterious in PsA. In our study, we reported no variation in the proportion of single IL-22 producing cells between untreated and MTX treated PsA patients. From our point of view, it is therefore important to evaluate the CD73 status of these IL-22 secreting cells to determine if they can be regulated by Ado. A recent study on the contrary suggests a regulatory role of IL-22 in PsA patients with a reported decreased IL-22^+^ cells frequency in PsA patients compared to HD [38]. However, in this paper, treated and untreated patients have been pooled together, making the interpretation more difficult. In RA, inflammation in the SF seems mediated by IL-17A independently of IL-22 signaling [39]. Nevertheless, IL-22 can be found in high concentrations in RA patients due to its production by Th17 and Th1.17 cells that are subsets highly active in the pathology.

In our study, MTX treatment of RA and PsA patients did not induce changes on Treg frequency and CD39 MFI (Figure S5C,E,F). This contrasts with another study showing increased CD39^+^ Treg frequencies in MTX responders [40]. However, in this study, the authors suggest the direct production of Ado by Treg expressing both CD39 and CD73 which is in contradiction with observations we and others have reported [12,41]. However, high CD39^+^ Treg frequency at baseline could correlate with good MTX response, and CD39 is therefore suggested as a biomarker of the MTX response in RA [40,42]. Our analysis of untreated and MTX-treated paired samples of RA and PsA patients demonstrated a CD73 frequency in Th subsets closer to HD in MTX-treated patients. This could result from an arrest in cell proliferation due to A2AR engagement by Ado released after MTX-induced AICAR blockade [15]. We hypothesize that restoration of CD73 expression, through degradation of AMP, in turn accentuates the Ado-mediated immunosuppression initiated by MTX. The loss of CD73 expression on Teff could indeed be necessary for their proliferation and pro-inflammatory features to escape CD39^+^ Treg-mediated suppression through cooperative Ado production.

In MTX-treated PsA patients, up-regulation of CD73 on Teff appears less important than in RA patients. This could rely on the fact that PsA is a joint affection mostly developing subsequently to established Pso, that is more heterogeneous in its characterization and symptoms. The immune-biological regulation of PsA inflammation is therefore different from RA and joint inflammation in PsA might be fueled by specific immune cells active in Pso, possibly interfering with the biological effect of MTX. Indeed, auto-reactive CD8^+^ T cells have been shown to contribute to Pso and should be considered. 

We also showed an enhanced cytokine production capacity of CD73^+^ Teff in RA patients after MTX treatment (IFN-γ, IL-17A, IL-17A/IFN-γ, IL-22). However, we can expect these cells to be less pro-inflammatory in vivo, since they express higher level of CD73, favoring Ado production. We and others have indeed previously showed that Ado strongly reduced CD73^+^ Teff cytokine pattern except IL-17A and, to a lesser extent, IL-22 [12,30]. These data suggest a benefit of MTX treatment, which may have synergistic effect with anti-IL-17A in contrast to TNF inhibitors in PsA [43]. Similarly, in RA, although combination of MTX and anti-IL-6R such as Tocilizumab has not shown clinically relevant short-term superiority over Tocilizumab monotherapy [44], concomitant MTX treatment with Tocilizumab may have an interest in low Tocilizumab responders first under monotherapy. Indeed, we evidenced down-regulation of IL-6R on CD73^+^ Teff compared to CD73^neg^ Teff (unpublished results), suggesting a reduced impact of anti-IL-6R on CD73^+^ Teff. Therefore, MTX could better neutralize activated and proliferating CD73^+^ Teff, while anti-IL-6R might target CD73^neg^ Teff; their combined action provide a better regulation of the overall hyper activated Teff population in the contexts of auto-immune disorders.

Purine metabolism and its regulation emerges as a pivotal regulator of immunity and inflammation, Modulation of this pathway in combination with MTX therefore appears as a promising target for new therapeutic strategies.

## Figures and Tables

**Table 1:** Characteristics of RA and PsA patients enrolled in the study.

	RA Patients *n* = 26	PsA Patients *n* = 15	Healthy Controls *n* = 12
Gender ratio (f/m, *n*)	19/7	9/6	8/4
Age (years)	56 (22–82)	53 (31–79)	49 (25–69)
DAS28 Untreated MTX-treated	4.6 (1.6–6.4) 3.4 (1.8–7.2)	-	
CRP (mg/mL) Untreated MTX-treated	17.9 (0.6–60) 5.2 (0.5–29)	16.4 (2.6–88) 4.3 (1.3–10)	
Mean MTX treatment duration (months)	9.3 (3–22)	6 (3–22)	-
MTX doses (mg/week)	(15–20)	(15–20)	-
RF detection (yes/no, *n*)	22/4	Not detected	-
ACPA detection (yes/no, *n*)	24/2	Not detected	-

Data presented as (range). RA. Rheumatoid Arthritis; PsA: Psoriatic Arthritis; DAS28: 28 joints activity score; RF: Rheumatoid Factor; ACPA: antibodies against cyclic citrullinated peptides. Two RA patients under MTX treatment also received cortancyl (7.5 mg).

## Supplementary Materials

The following are available online at https://www.mdpi.com/2077-0383/8/11/1859/s1, Figure S1: Co analysis of phenotypic and functional characteristics of Th subsets; Figure S2: Altered frequencies of CD73+ T cells in patients blood reflecting their high activation; Figure S3: Correlation between proportion of CD39+ Treg, CD73 on Th1, Th17 and Th1.17 and disease activity score (DAS28) in untreated RA patients; Figure S4: Teff from RA and PsA patients evidence the same capacities for cytokine secretion but an increase in Th22 cells is observed in peripheral blood of PsA patients; Figure S5: MTX treatment induces a slight decrease of memory CD8+ T cells frequency in PsA patients and does not modify Treg proportion and activation status in RA and PsA patients; Figure S6: Th1.17 cells are the major producers of Ado among Teff in a CD73 dependent manner; Figure S7: MTX treatment moderately increase IL-17A secretion by Teff in PsA but not in RA patients; Table S1: Panels of antibodies used for multi-parametric flow cytometry analysis throughout the study.
