# Supplementary material for: Methotrexate Restores CD73 Expression on Th1.17 in Rheumatoid Arthritis and Psoriatic Arthritis Patients and May Contribute to Its Anti-Inflammatory Effect through Ado Production
Source: J Clin Med. 2019 Nov 3;8(11):1859. doi: 10.3390/jcm8111859 (PMC6912794; doi:10.3390/jcm8111859)
Supplement: Supplementary file 1 [file jcm-08-01859-s001.pdf]

Supplementary Materials

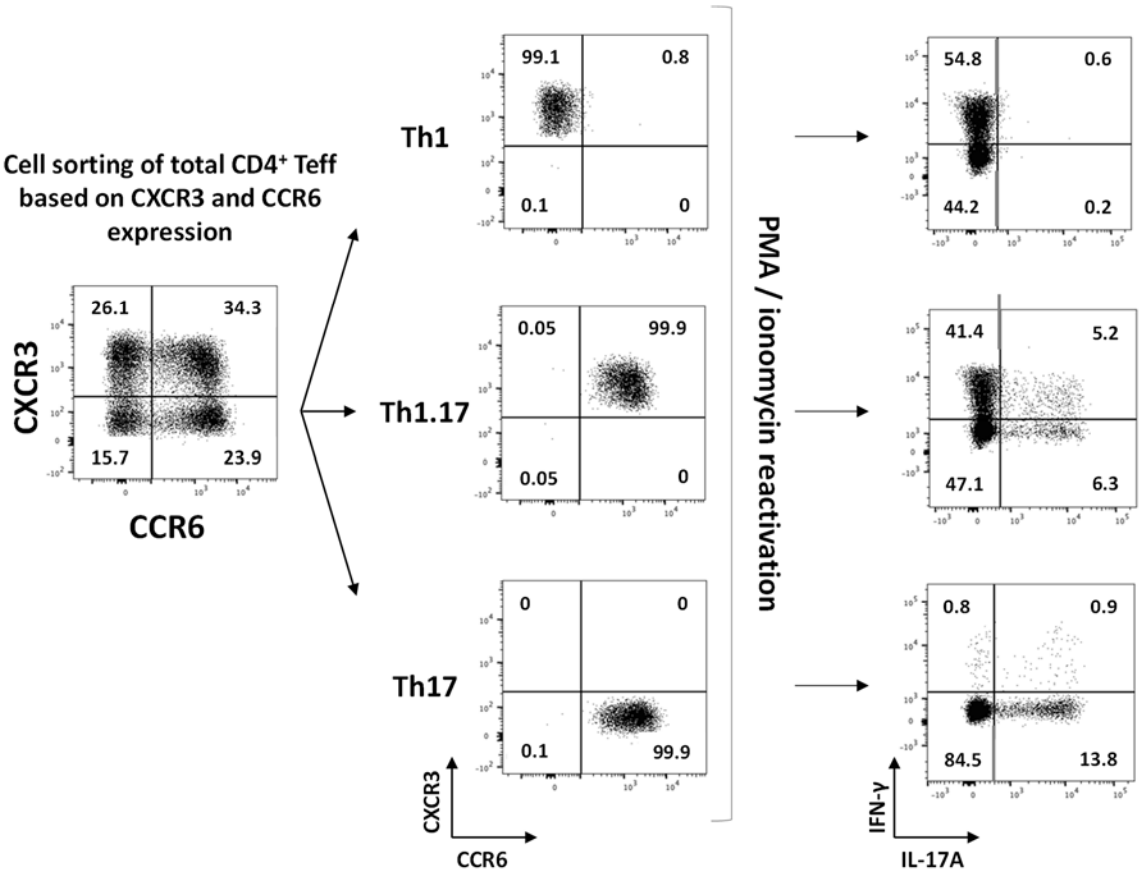

Supplementary Figure S1. Co-analysis of phenotypic and functional characteristics of Th subsets. Th1, Th1.17 and Th17 subpopulations based on CXR3 and CCR6 expression were sorted from total memory CD4⁺ T cells and IL-17A and IFN-γ production capacity was analyzed after short term reactivation with PMA-Ionomycin.

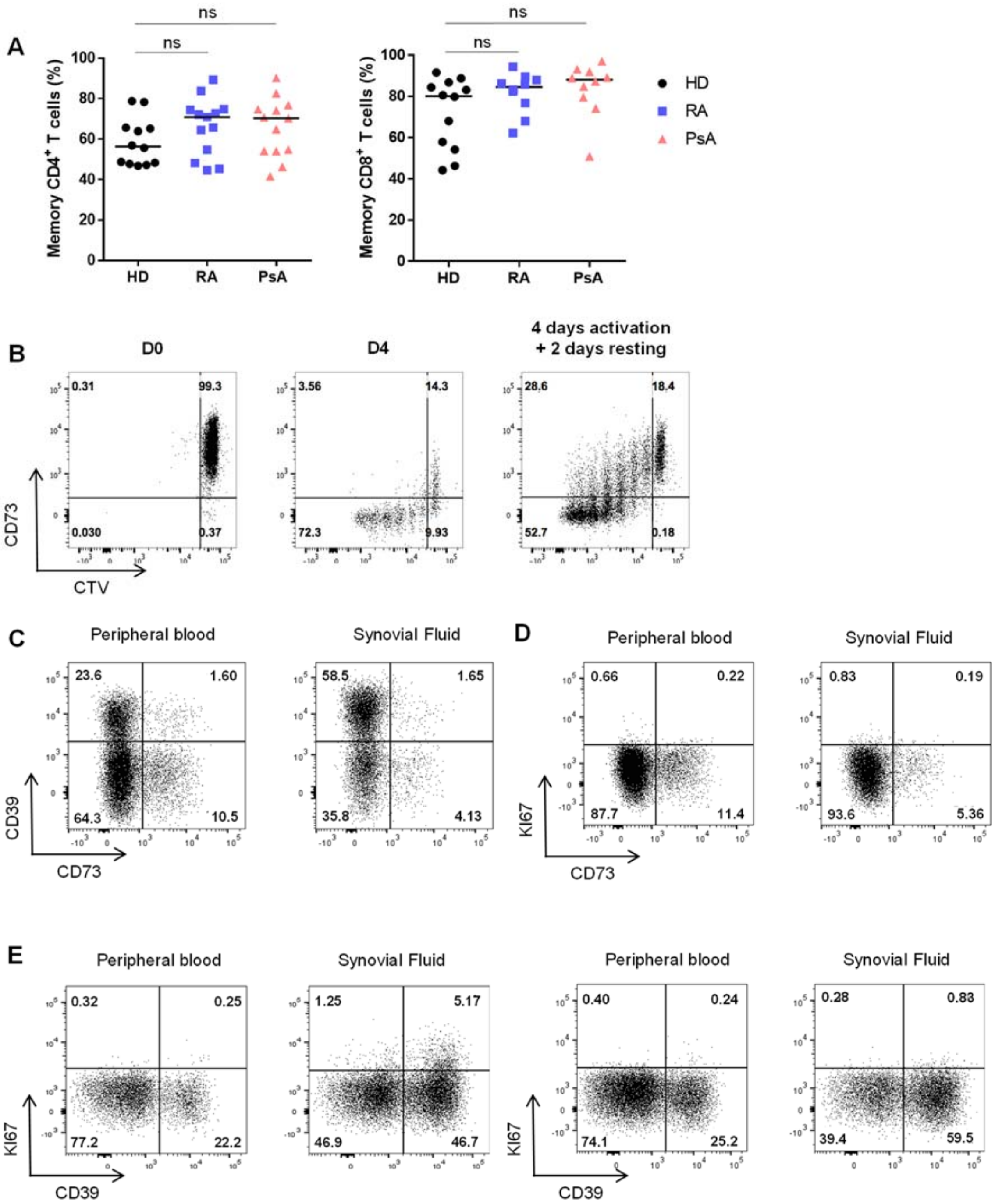

Supplementary Figure S2 The overall frequencies of memory CD4+ and CD8+ T cells are not altered in patients' blood, however the frequency of memory CD73+ T cells is decreased in both compartments reflecting their high level of activation. (**A**): Memory CD4+ (**left**) and memory CD8+ (**right**) frequencies in HD, untreated RA and PsA patients' peripheral blood. (**B**): CD73 dynamic of expression assessed by FC on sorted CD73+ blood Teff stimulated in vitro with anti-CD3/anti-CD28 coated beads for 96h. (**C**,**D**): CD73/CD39 (**C**) and CD73/Ki67 (**D**) staining on total Teff in peripheral blood and synovial fluid of a PsA patient before initiation of treatment. (**E**,**F**): KI67/CD39 staining on total Teff from peripheral blood orSF of RA (**E**) and PsA (**F**) patients before initiation of treatment. Statistics A Kruskal-Wallis test.

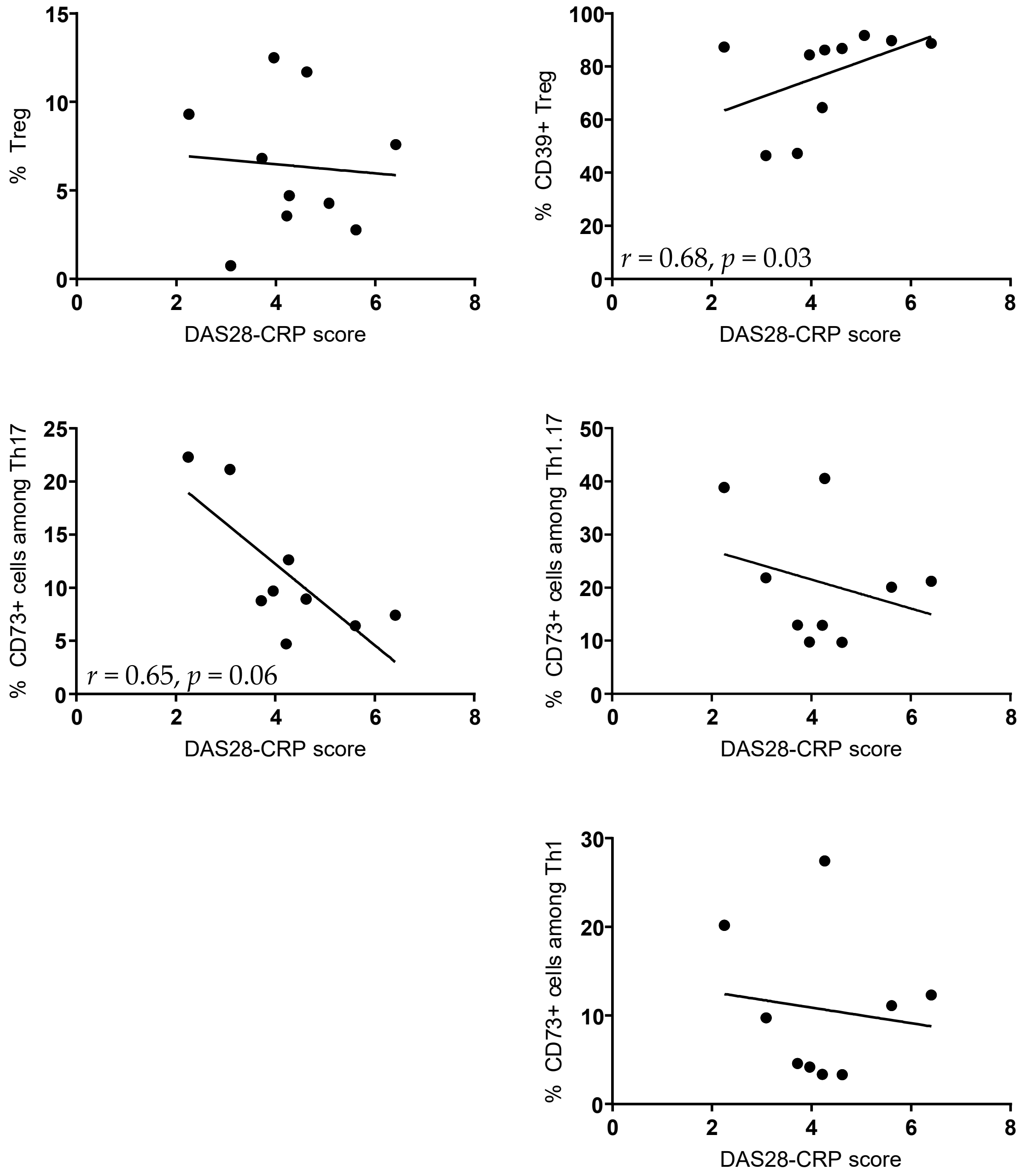

Supplementary Figure S3. Correlation between proportion of CD39 among Treg, and CD73 on Th1, Th17 and Th1.17 and clinical disease activity (DAS28-CRP) in untreated RA patients. Correlations were calculated using Spearman's test.

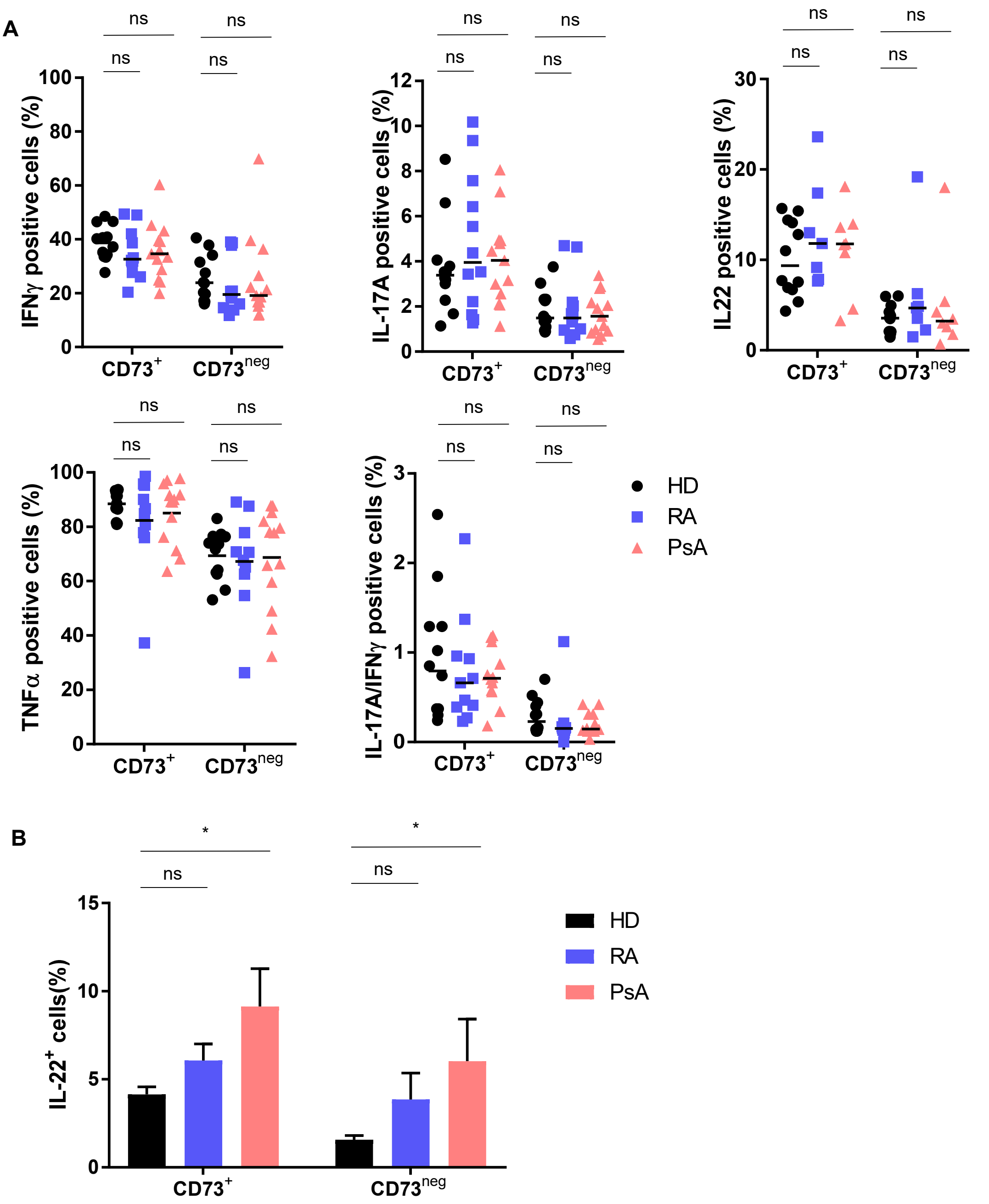

Supplementary Figure S4. Teff from RA and PsA patients evidence the same capacities for cytokine secretion but an increase in Th22 cells is observed in peripheral blood of PsA patients. **A**: Monitoring of IFN-γ, IL-17A, IL-22, TNF-α producing and IFN-γ/IL-17A co-producing Teff stratified on their CD73 expression. **B**: Analysis of single IL-22 producing Teff in HD and untreated RA and PsA patients. **A**,**B**: ANOVA-2. * $p < 0.05$.

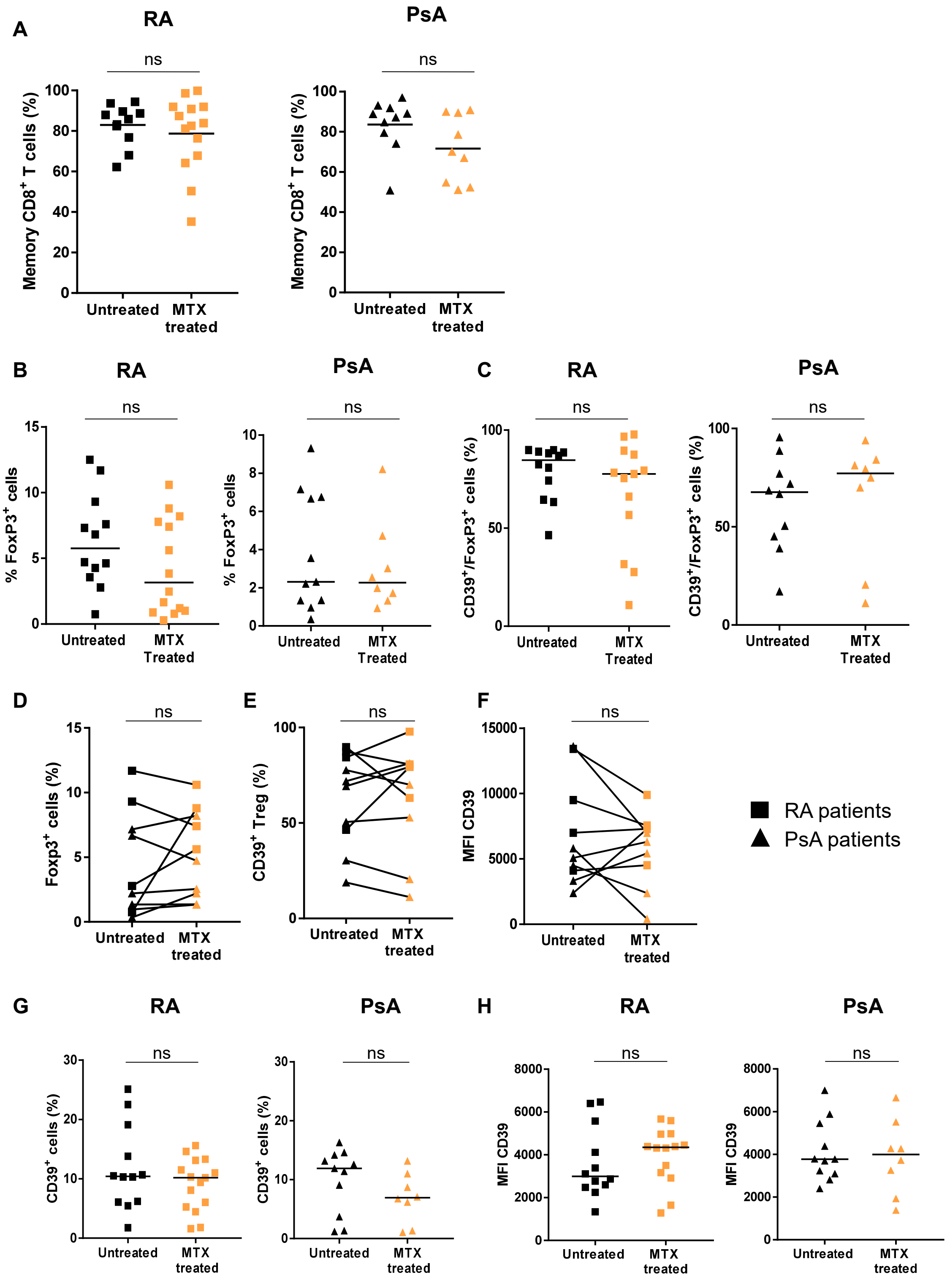

Supplementary Figure S5. MTX treatment induces a slight decrease of memory CD8⁺ T cells frequency in PsA patients and does not modify Treg proportion and activation status in RA and PsA patients. **A**: Memory CD8⁺ T cells frequencies in untreated vs MTX-treated RA (**left**) and PsA (**right**) patients. **B**: Frequencies of FoxP3⁺ Treg in untreated vs. MTX-treated RA (**left**) and PsA (**right**) patients. **C**. Frequencies of CD39⁺ cells among Treg in untreated vs. MTX-treated RA (**left**) and PsA (**right**) patients. **D–F**: Frequencies of FoxP3⁺ cells (**D**), CD39⁺ Treg (**E**) and MFI of CD39 on Treg (**F**) in paired samples of untreated vs. MTX-treated RA and PsA patients. **G,H**: Frequencies (**G**) and MFI (**H**) of CD39⁺ Teff in RA or PsA patients before or under MTX treatment. **A–C** and **G,H**: Mann-Whitney test, **D–F**: Wilcoxon test.

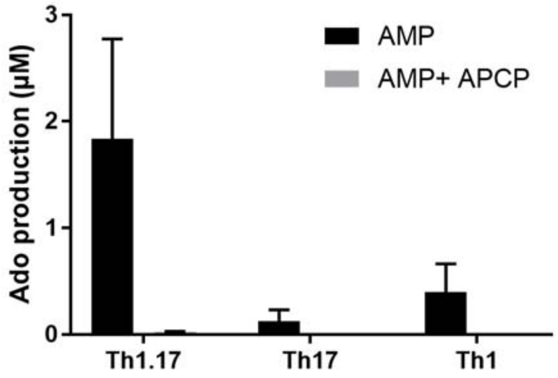

Supplementary Figure S6. Th1.17 cells are the major producers of Ado among Teff in a CD73 dependent manner. Ado produced by sorted Th1.17, Th17 and Th1 subsets from Healthy donors (n = 3). Cells were incubated for 2 hours with AMP13C15N isotope (37.5 µM) +/− APCP (50 µM) before Ado quantification in supernatants by LC-MS/MS.

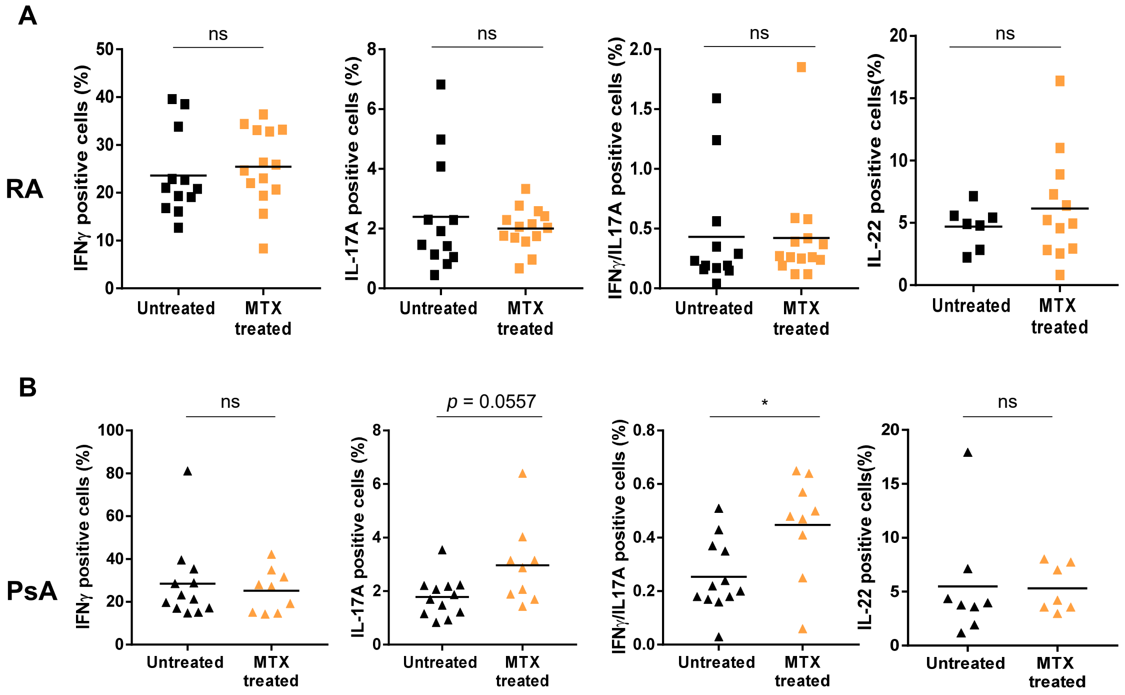

Supplementary Figure S7. MTX Moderately increases of IL-17A secretion by Teff in PsA but not in RA patients. Analysis of IFN-γ, IL-17A, IL-22 producing and IFN-γ/IL-17A co-producing Teff in RA (**A**) and PsA (**B**) patients before treatment initiation and under MTX treatment. * $p < 0.05$, assessed by Mann-Whitney test.

Supplementary Table S1. Panels of antibodies used for multi-parametric flow cytometry analysis throughout the study.

Panel A	Panel B	Panel C	Panel D
CD3 (UCHT1) BD Biosciences	CD3 (UCHT1) BD Biosciences	CD3 (UCHT1) BD Biosciences	CD45RA (2H4LDH11LDB9) Beckman Coulter
CCR7 (150503) BD Biosciences	CD45RA (HI100) Biolegend	CD4 (RPA-T4) Life Technologies	CD127 (M21) Life Technologies
CD45RA (HI100) Biolegend	CCR6 (11A9) BD Biosciences	CD45RA (HI100) Biolegend	CD25 (2A3) BD Biosciences
CD8 (RPA-T8) Beckman Coulter	CXCR3 (REA232) Miltenyi-Biotec	IL-17A (N49-653) BD Biosciences	CCR6 (11A9) BD Biosciences
CD4 (RPA-T4) Life Technologies	CD4 (RPA-T4) Life Technologies	TNF-α (MAb11) BD Biosciences	CXCR3 (REA232) Miltenyi-Biotec
CD39 (A1) Life Technologies	CD39 (A1) Life Technologies	IFN-γ (4S. B3), Biolegend	DAPI Life Technologies
CD73 (AD2) Life Technologies	CD73 (AD2) Life Technologies	IL-22 (22URTI) Life Technologies	
FoxP3 (PCH01) Life Technologies	FoxP3 (PCH01) Life Technologies	CD73 (AD2) Life Technologies	
